# Comparative assessment of extravascular lung water index in ARDS patients on veno-venous ECMO: transpulmonary thermodilution versus AI-driven chest CT segmentation

**DOI:** 10.1186/s12890-026-04260-9

**Published:** 2026-04-07

**Authors:** Matthias Otto, David Mohr, Julia Zimmermann, Nils Rathmann, Christoph Boesing, Manfred Thiel, Joerg Krebs, Thomas Luecke, Patricia R.M. Rocco, Alice Marguerite Conrad

**Affiliations:** 1 Department of Anesthesiology and Critical Care Medicine, Faculty of Medicine, University Hospital Mannheim, University of Heidelberg, Theodor-Kutzer Ufer 1-3, Mannheim, 68165 Germany; 2 Department of Clinical Radiology and Nuclear Medicine, Faculty of Medicine, University Hospital Mannheim, University of Heidelberg, Theodor-Kutzer Ufer 1-3, Mannheim, 68165 Germany; 3 Laboratory of Pulmonary Investigation, Carlos Chagas Filho Institute of Biophysics, Centro de Ciências da Saúde, Federal University of Rio de Janeiro, Avenida Carlos Chagas Filho, 373, Bloco G-014, Ilha do Fundão, Rio de Janeiro, Brazil; 4 Department of Anesthesiology and Critical Care Medicine, Faculty of Medicine, University Hospital Mannheim, University of Heidelberg, Theodor-Kutzer-Ufer 1-3, Mannheim, D-68167 Germany

**Keywords:** Acute respiratory distress syndrome, Pulmonary edema, Transpulmonary thermodilution, Extravascular lung water index, Automated lung segmentation, Computed tomography, Extracorporeal membrane oxygenation

## Abstract

**Background:**

In severe acute respiratory distress syndrome (ARDS) patients on veno-venous extracorporeal membrane oxygenation (VV ECMO), restrictive fluid management reduces pulmonary edema, quantified by extravascular lung water index (EVLWI), and improves outcomes. Bedside EVLWI is commonly assessed using single-indicator transpulmonary thermodilution (TPTD), which is increasingly employed to guide fluid therapy. However, TPTD-derived EVLWI values can be significantly affected by extracorporeal blood flow (ECBF), leading to inaccurate estimates during ECMO. In contrast, artificial intelligence (AI)-based automated lung segmentation of chest computed tomography (CT) enables reliable ECBF-independent visualization and quantification of pulmonary edema (EVLWI_CT_). The primary objective was to compare EVLWI measured by TPTD (EVLWI_TPTD_) with EVLWI_CT_ in ARDS patients on VV ECMO. The secondary objective was to derive and validate an ECBF-dependent correction factor to improve the accuracy of EVLWI_TPTD_.

**Methods:**

In this retrospective, single-center observational cohort study of 64 patients with severe ARDS on VV ECMO, routine chest CT images and concurrent TPTD measurements, performed immediately before or after the CT scan, were analyzed. EVLWI was quantified using AI-based automated CT segmentation (EVLWI_CT_) and TPTD (EVLWI_TPTD_), the latter performed immediately before or after the CT scan. A multiple regression model using EVLWI_CT_ as the reference was applied to derive an ECBF-dependent correction factor for EVLWI_TPTD_ (EVLWI_TPTDcorr_).

**Results:**

EVLWI_TPTD_ significantly overestimated pulmonary edema compared to EVLWI_CT_ (24.3 [15.8–30.0] vs. 10.2 [5.8–14.8] ml/kg; *p* < 0.001), with a mean bias of − 12.3 ml/kg and limits of agreement from − 30.9 to 6.2 ml/kg. A correction factor of 3.439 ml/kg per liter of ECBF was identified. After adjustment, EVLWI_TPTDcorr_ did not differ significantly from EVLWI_CT_ (8.7 [4.2–15.8] vs. 10.2 [5.8–14.8] ml/kg; *p* = 0.393), with a reduced bias of 0.96 ml/kg and narrower limits of agreement (–16.4 to 18.4 ml/kg).

**Conclusion:**

In severe ARDS patients on VV ECMO, EVLWI obtained via TPTD was significantly overestimated due to ECBF interference. TPTD systematically overestimates EVLWI due to ECBF interference. Incorporating an ECBF-adjusted correction factor markedly improves agreement with AI-based CT quantification, enhancing the reliability and clinical applicability of TPTD-derived EVLWI for fluid management in this high-risk population.

**Trial registration:**

German Clinical Trials Register (DRKS00026246). Registered 14/09/2021. https://drks.de/search/en/trial/DRKS00026246.

**Supplementary Information:**

The online version contains supplementary material available at 10.1186/s12890-026-04260-9.

## Background

Acute respiratory distress syndrome (ARDS) is characterized by diffuse alveolar damage and excessive fluid accumulation within the lungs [1], leading to pulmonary edema and increased tissue density, especially in gravity-dependent lung regions. This edema contributes to alveolar collapse, impaired gas-exchange, and progressive respiratory failure [2, 3]. In mechanically ventilated ARDS patients, the extravascular lung water index (EVLWI), derived from single-indicator transpulmonary thermodilution (TPTD), is a widely used and validated bedside method to quantify pulmonary edema and assess the severity of lung injury [4–6]. EVLWI_TPTD_ is increasingly employed to guide fluid management in critically ill patients, including those with ARDS [4–6].

In patients with severe ARDS, who fail to respond to conventional lung-protective ventilation, veno-venous extracorporeal membrane oxygenation (VV ECMO) has been shown to improve survival [7, 8]. In these patients, a restrictive fluid management minimizing pulmonary edema is considered essential and has been associated with better outcomes [9–11]. However, the clinical utility of TPTD in VV ECMO patients is limited by the confounding effect of extracorporeal blood flow (ECBF), which can result in significant overestimation of EVLWI and inaccurate assessment of fluid status [12, 13].

Chest computed tomography (CT) is the reference imaging modality for detecting and characterizing pulmonary edema [14], but quantitative EVLWI assessment through CT has been underutilized due to the time-consuming and expertise-dependent process of manual lung segmentation [15]. Recent advances in artificial intelligence (AI) now allow automated lung segmentation, enabling rapid and reproducible quantification of CT-derived extravascular lung water (EVLWI_CT_) in ARDS patients without VV ECMO [16]. Notably, this approach is independent of ECMO-related hemodynamic interference and could serve as a more reliable tool for assessing lung water burden.

Therefore, the primary objective of this study was to compare EVLWI values obtained from AI-based automated CT segmentation (EVLWI_CT_) with those derived from transpulmonary thermodilution (EVLWI_TPTD_). The secondary objective was to derive and validate an ECBF-dependent correction factor to improve the accuracy and bedside applicability of EVLWI_TPTD_ in this patient population.

## Methods

### Ethical approval

This retrospective, observational, single-center cohort study was conducted of full compliance with the Declaration of Helsinki in the 25-bed intensive care unit of the Department of Anesthesiology and Critical Care Medicine, University Hospital Mannheim. Ethical approval was obtained from the local Institutional Review Board (Medizinische Ethikkommission II, University Medical Centre Mannheim, Medical Faculty Mannheim of the University of Heidelberg, Mannheim, registration number 2021 − 831). Due to the retrospective observational nature of the study, the need for consent from the participants of the study was waived by the Institutional Review Board. The study was registered in the German Clinical Trials Register (DRKS00026246, https://drks.de/search/en/trial/DRKS00026246) prior to data analysis. A detailed institutional protocol for the management of severe ARDS patients on VV ECMO and TPTD monitoring is available in the Additional Files.

### Inclusion and exclusion criteria

Severe ARDS patients on VV ECMO who underwent chest computed tomography (CT) with simultaneous TPTD monitoring between January 2016 and December 2021 were eligible. ARDS was defined according to the relevant guidelines [17]. The indication for VV ECMO therapy was determined at the discretion of the attending physician. CT scans with contrast agents were not excluded [18], nor were patients with pleural effusion or pneumothorax. To prevent repeated measurements, only the first available CT scan per patient was included in the analysis. To minimize the potential influence of changes in fluid status, only patients with TPTD measurements performed within six hours of CT imaging were included.

### Clinical data acquisition

Anthropometric and clinical characteristics were extracted from the electronic patient data management system (Philips Intelli Space Critical Care and Anesthesia).

### Chest computed tomography scan protocol

All chest CT scans included in this study were performed according to the standardized imaging protocols of the Department of Clinical Radiology and Nuclear Medicine, University Hospital Mannheim, under the supervision of a board-certified radiology consultant. All image analyses were supervised by a senior radiologist (NR) to ensure methodological accuracy and to confirm exclusion of pleural effusions and pneumothorax from the final segmentation dataset. Chest CT scans were performed using a second-generation dual source CT scanner (Somatom Definition Flash, Siemens Healthineers). Acquisition parameters included: 32 × 0.6 mm collimation, a slice thickness of 1.5 mm, increment of 1.2 mm, and medium-soft convolution kernels (I31f; Q33f 89/76), reference mAs at 120 kV, pitch of 0.8, and rotation time of 0.5 s.

### Quantification of EVLWI from CT scans

Retrospective image analysis of chest CT scans was conducted with 3D Slicer (http://www.slicer.org), an open-source medical imaging platform. Voxel density was measured in Hounsfield units (HU). Automated lung segmentation was conducted using the Lung CT Segmenter tool from the Chest Imaging Platform, employing the R-231 model [15], as previously described by our group in non-ECMO patients [16]. Only voxels with HU values associated with pulmonary edema (− 700 to 200 HU) were included [19, 20], while voxels corresponding to contrast agents (> 200 HU) were excluded [21, 22]. This segmentation approach follows the protocol previously validated by our group [16]. Lung weight was calculated using the following formula [23]:

$$\begin{array}{ll}\mathrm{Voxel}\;\mathrm{tissue}\;\mathrm{weight}\\=\left(1-\left(\mathrm{Voxel}\;\mathrm{density}-1000\right)/1000\right)\\\times\mathrm{Voxel}\;\mathrm{volume}\end{array}$$

resulting in:

$$\begin{array}{ll}\mathrm{Lung}\;\mathrm{weight}\\=\left(1-\left(\mathrm{Mean}\;\mathrm{HU}\right/-1000\right))\\\times\mathrm{Lung}\;\mathrm{volume}\end{array}$$

Expected lung weight was calculated using the formula by Cressoni et al. [24]

$$\begin{array}{ll}\mathrm{Expected}\;\mathrm{lung}\;\mathrm{weight}\;\left(\mathrm g\right)\\=-1806.1+1633.7\times\mathrm{height}\left(\mathrm m\right)\end{array}$$

Assuming that 1 mg of excess lung tissue corresponds to 1 ml of extravascular lung water, EVLWI derived from the CT analysis was normalized to ideal body weight (IBW):

$$\begin{array}{ll}\mathrm{EVLWI}_\mathrm{CT}\mathrm{[ml/kg]} =\\ \left(\text{Calculated lung weight - Expected lung weight}\right)\\/\mathrm{IBW}\end{array}$$

The accuracy of the automated lung segmentation was evaluated using a three-point grading system [16]. For comparison, a manual segmentation of each CT scan was also performed by an experienced intensivist using the Segment Editor in 3D Slicer, with the EVLWI from manual segmentation (EVLWI_CTman_​) calculated using the same approach. All manual analyses were performed by a senior physician in radiology.

### Quantification of EVLWI using TPTD

Data for EVLWI_TPTD_ was extracted from electronic medical records. The TPTD measurement closest in time to the CT scan was selected for analysis.

### Correction of TPTD-derived EVLWI for ECBF

To account for the influence of extracorporeal blood flow, a multiple linear regression analysis was performed using EVLWI_CT_ as the reference variable. The resulting correction model (EVLWI_TPTDcorr_) was applied as follows:

$$\begin{aligned}&\mathrm{EVLWI}_\mathrm{TPTDcorr}\;\lbrack\mathrm{ml}/\mathrm{kg}\rbrack\;\\&=\left({\mathrm{EVLWI}}_{\mathrm{TPTD}}\right. \\& \left. -\left(Correction\mathit\;Factor\;\mathrm x\;\mathrm{ECBF}\right)\right)\end{aligned}$$

The full analysis workflow is illustrated in Additional Files (Figure S1).

### Statistical analysis

Sample size estimation was based on pilot data from our previous study [16], which compared CT-derived and transpulmonary thermodilution–derived EVLWI measurements in 145 ARDS patients without ECMO using the same institutional protocol and equipment. The standard deviation of paired differences was derived from the reported limits of agreement (530.6 ml), corresponding to 7.90 ml/kg of ideal body weight. Using the precision-based Bland–Altman method [25], a minimum of 45 patients was required to estimate each limit of agreement with a 95% confidence interval half-width of ± 4 ml/kg. For the primary paired comparison, a sample size of 20 patients provided 80% power (two-sided α = 0.05) to detect a mean difference of 5 ml/kg between EVLWI_CT_ and EVLWI_TPTD_. Given the anticipated additional variability in ECMO patients due to extracorporeal blood flow interference, we predefined a target sample size of at least 60 patients. The final cohort of 64 patients therefore fulfilled these criteria, providing an expected precision of ± 3.4 ml/kg for the limits of agreement and 80% power to detect a mean difference between EVLWI_CT_ and EVLWI_TPTD_ of ≥ 2.8 ml/kg.

The distribution of EVLWI_CT_ and EVLWI_TPTD_ values was assessed using Shapiro- Wilk test. Comparisons between groups were performed using a paired T-test for normally distributed data and Wilcoxon rank test for non-parametric data. The relationship between EVLWI_CT_ and EVLWI_TPTD_ was further evaluated using Spearman correlation analysis.

Agreement between measurement methods was evaluated using Bland–Altman analysis, with bias defined as the mean difference and 95% limits of agreement (LoA) calculated as the mean difference ± 1.96 × standard deviation [26]. Following ECBF correction, EVLWI_CT_ and EVLWI_TPTDcorr_ were compared using the same statistical approach. The DICE similarity coefficient was computed to assess agreement between automated and manual CT segmentations, as previously described [27].

Continuous variables were presented as mean ± standard deviation (SD) for normally distributed data, and as median with first and third quartiles (Q1–Q3) for non-normally distributed data. A p-value < 0.05 was considered statistically significant. All analyses were performed using GraphPad Prism version 8.0.2 (GraphPad Software, San Diego, CA, USA).

## Results

Between January 2016 and December 2021, 64 patients with severe ARDS on VV ECMO underwent simultaneous chest CT scans and hemodynamic monitoring with TPTD. Anthropometric data and baseline clinical characteristics of the study population are summarized in Table 1, while detailed data on respiratory mechanics, gas exchange, ventilator settings, advanced hemodynamics, and laboratory parameters are provided in Additional Files (Tables S1-S3)).

**Table 1:** Anthropometric and Clinical Characteristics of the Study Population on the day of the Chest CT-Scan Acquisition

Age [years]	Study population (*n* = 64)
	53 ± 14
Female [%]	23
Body weight [kg]	94 ± 25
Height [cm]	177 ± 9
Body mass index [kg/m^2^]	30 ± 7
SAPS II score at admission	65 ± 11
ICU length of stay [days]	25 ± 22
Time between CT scan and TPTD measurement [hours]	2 (1–4)
Hemodialysis/CRRT [%]	38
Cumulative fluid balance since ICU admission [ml]	0 (−395–1514)
Extracorporeal blood flow rate at the time of CT scan [l/min]	3.9 ± 1.1
ECMO gas flow at the time of CT scan [l/min]	2.9 ± 1.4

Data are presented as mean ± SD, or median (interquartile range). *CTs *implified acute physiology score,*ICU *Intensive care unit,*CRRT *Continuous renal replacement therapy

The median time interval between CT acquisition and TPTD measurement was 2 h (IQR, 1–4 h), during which both ventilator and ECMO settings remained unchanged.

**Fig. 1:**
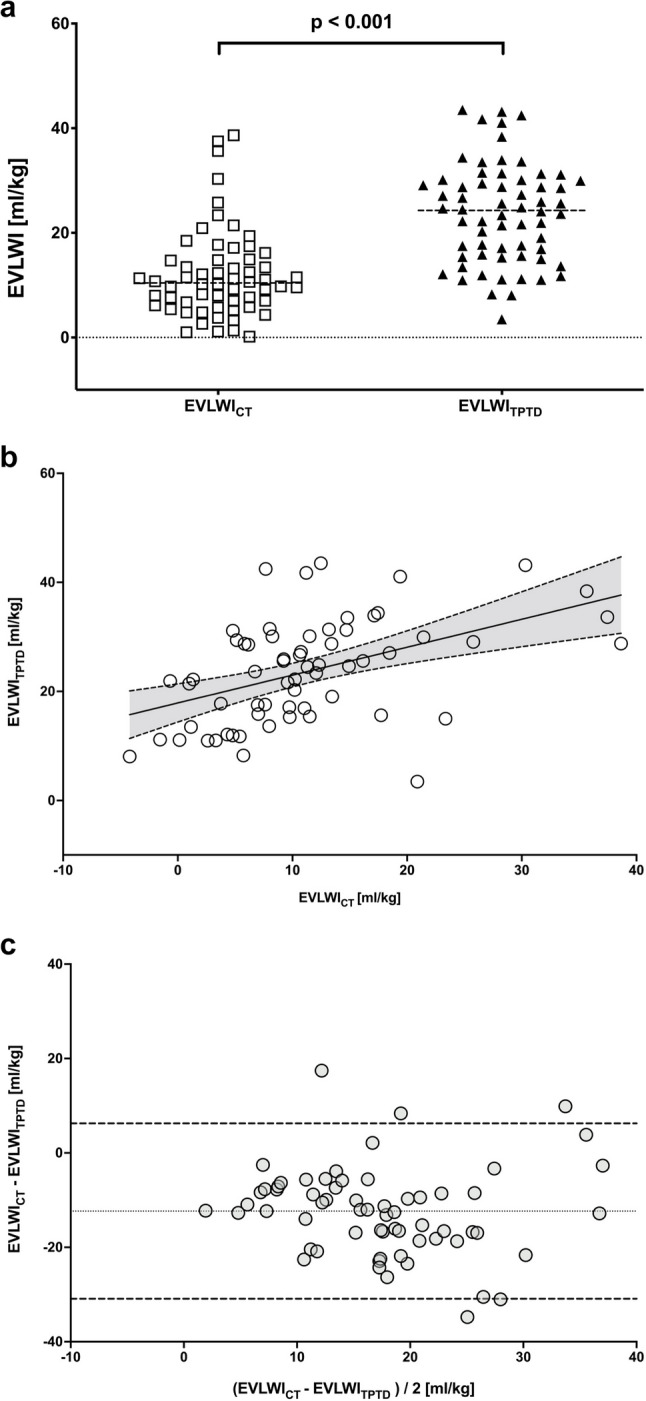
**a** Extravascular lung water index (EVLWI) in severe ARDS patients on VV ECMO, quantified artificial intelligence–based, automated chest CT segmentation (EVLWI_CT_) and transpulmonary thermodilution (EVLWI_TPTD_). The scatter dot plot shows the distribution range of EVLWI values for each method. Horizontal dashed lines indicate the median values. Statistical differences between the measurement modalities are indicated by brackets. **b **Spearman correlation analysis of extravascular lung water index (EVLWI) quantified by artificial intelligence–based, automated chest CT segmentation (EVLWI_CT_) and transpulmonary thermodilution (EVLWI_TPTD_). A moderate correlation (r = 0.47; p <0.001) with the regression equation: y = 0.51× *x* + 18 was observed. Dashed lines represent the 95% confidence interval. **c** Bland-Altmann plot comparing extravascular lung water index quantified by artificial intelligence–based, automated chest CT segmentation (EVLWI_CT_) and transpulmonary thermodilution (EVLWI_TPTD_). The dotted line represents the mean bias (-12.33 ml/kg), while the dashed lines show the limits of agreement (LOA): lower LOA at -30.91 ml/kg and upper LOA at 6.26 ml/kg

EVLWI_TPTD_ was significantly higher than EVLWI_CT_ (24.3 (15.8–30) vs. 10.2 (5.8–14.8) ml/kg, *p* < 0.001) (Fig. 1a). Despite this overestimation, a moderate but significant correlation was observed between the two modalities (*r* = 0.47; *p* < 0.001) (Fig. 1b). Bland-Altman analysis revealed a mean bias of − 12.3 ml/kg with limits of agreement ranging from − 30.9 to 6.3 ml/kg (Fig. 1c).

To account for the confounding influence of ECBF on EVLWI_TPTD_, a multiple regression model was constructed, yielding the following regression formula.

$$\begin{array}{ll}\mathrm{EVLWI}_\mathrm{TPTD}\;\left(\mathrm{ml}/\mathrm{kg}\right)\\=5.054+0.4711\\\times{\mathrm{EVLWI}}_{\mathrm{CT}}\\+3.439\times\mathrm{ECBF}\end{array}$$

**Fig. 2:**
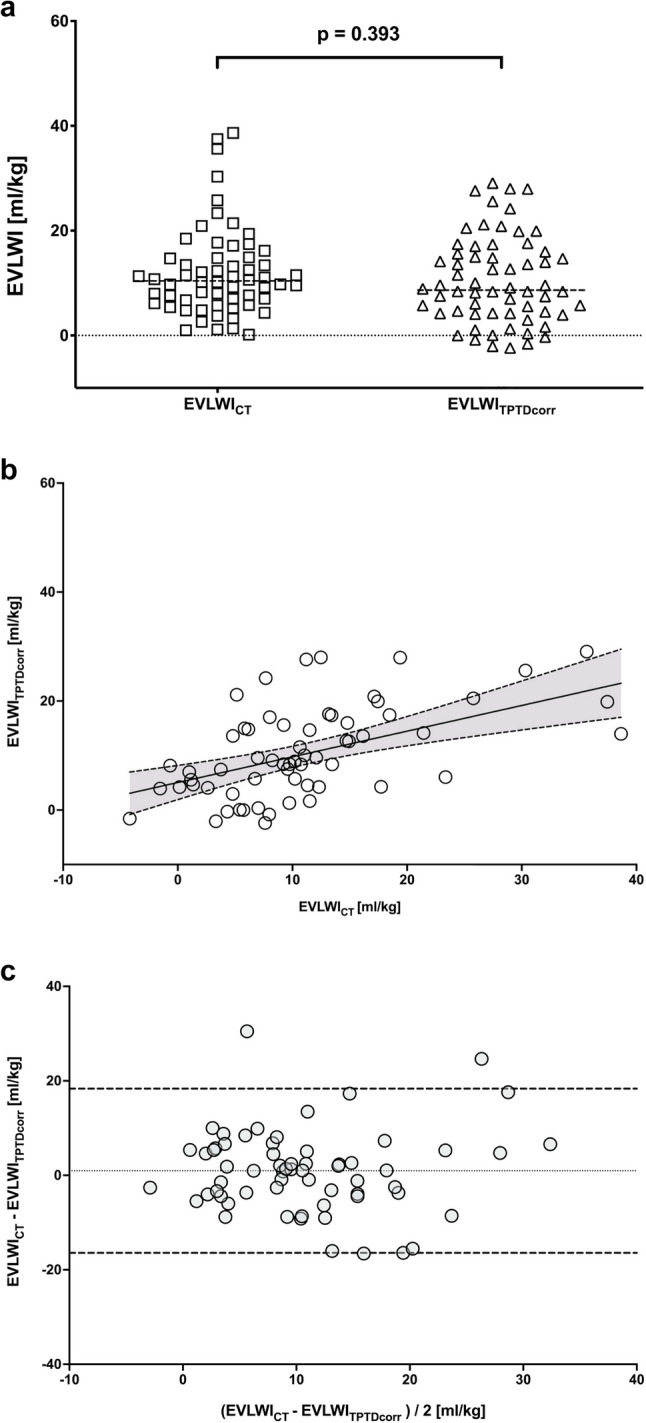
**a** Extravascular lung water index (EVLWI) in severe ARDS patients on VV ECMO, quantified by artificial intelligence–based, automated chest CT segmentation (EVLWI_CT_) and transpulmonary thermodilution corrected for extracorporeal membrane oxygenation blood flow (EVLWI_TPTDcorr_). The scatter dot plot shows the distribution of values for each method. Horizontal dashed lines represent medians; brackets indicate statistically significant differences between modalities. **b **Spearman correlation analysis of extravascular lung water index (EVLWI) quantified by artificial intelligence–based, automated chest CT segmentation (EVLWI_CT_) and transpulmonary thermodilution corrected for extracorporeal membrane oxygenation blood flow (EVLWI_TPTDcorr_). A significant correlation (r = 0.48, p < 0.001) with the regression equation: *y*= 0.4711 × *x* + 5.053 was observed. Dashed lines indicate the 95% confidence interval. **c **Bland-Altmann plot comparing extravascular lung water index quantified by artificial intelligence–based, automated chest CT segmentation (EVLWI_CT_) and transpulmonary thermodilution corrected for extracorporeal membrane oxygenation blood flow (EVLWI_TPTDcorr_). The dotted line shows the mean bias (0.96 ml/kg), while dashed lines represent the limits of agreement: lower LOA –16.43 ml/kg, upper LOA 18.35 ml/kg

After applying this correction, EVLWI_TPTDcorr_ values were no longer significantly different from EVLWI_CT_ (8.7 [4.2–15.8] vs. 10.2 [5.8–14.8] ml/kg, *p* = 0.393; Fig. 2a). The correlation between the two measures remained significant (*r* = 0.48, *p* < 0.001; Fig. 2b). Bland-Altman analysis showed a reduced mean bias (0.96 ml/kg) and narrower limits of agreement (–16.4 to 18.4 ml/kg; Fig. 2c), indicating improved concordance between corrected TPTD- and CT-derived EVLWI. The influence of ECBF on EVLWI_TPTD_ relative to EVLWI_CT_ is detailed in Additional File Table S4. A comparison between manual versus AI-based automated CT segmentation approaches is presented in Figures S2–S4. Regarding the performance of automated lung segmentation, image quality was rated as good in 30 patients (47%), moderate in 20 (31%), and poor in 14 patients (22%) using a standardized three-point grading system. The DICE similarity coefficient comparing automatically and manually segmented lung volumes was 0.973, indicating excellent spatial agreement.

## Discussion

We conducted a retrospective, observational, single-center cohort study comparing EVLWI quantified using AI-based automated chest CT scan segmentation, which is unaffected by ECBF, and TPTD in severe ARDS patients on VV ECMO. Furthermore, we calculated a correction factor incorporating ECBF to improve the clinical utility of TPTD-derived EVLWI.

In our study, uncorrected EVLWI_TPTD_ values were significantly higher than EVLWI_CT_. Although the two methods showed a statistically significant correlation, Bland-Altman analysis showed a high bias and wide limits of agreement. Incorporating ECBF into a regression model yielded a correction factor that substantially reduced bias and improved agreement with EVLWI_CT_, highlighting a practical adjustment to improve bedside monitoring. Accurate quantification of pulmonary edema is critical in managing ARDS patients, both with and without VV ECMO, as EVLWI serves as a prognostic marker [11, 28] and guides therapeutic decision-making [29, 30]. In clinical practice, TPTD is widely used outside ECMO; however, its reliability is compromised in VV ECMO due to interference from ECBF [12, 13]. Conversely, chest CT is not influenced by ECBF and may offer a robust alternative by quantifying lung tissue and gas volumes [16]. Yet, manual CT segmentation is labor-intensive, prone to misclassification, and clinically impractical [31]. Advances in artificial intelligence now permit rapid, fully automated segmentation with high reproducibility, as supported by our data showing excellent concordance between automated and manual lung volumes (Dice coefficient 0.973). These technological advances may help reintroduce CT-based lung water quantification into clinical practice [31].

Our findings align with prior reports indicating that VV ECMO modifies thermodilution curves by prolonging downslope times due to thermoindicator recirculation within the extracorporeal circuit [12, 13, 32]. This phenomenon parallels the effects seen in extracorporeal therapies such as hemofiltration [33, 34]. Previous studies suggested a correction factor of ~ 3.0 ml/kg per liter of ECBF [13]; our regression analysis yielded a similar coefficient of 3.439 ml/kg, reinforcing both the physiological rationale and reproducibility of this adjustment. From a clinical perspective, overestimation of EVLWI may mislead fluid management strategies in this fragile population. Both fluid overload [35] and excessive deresuscitation [36] are modifiable risk factors in ARDS patients on ECMO. Thus, a multimodal approach to volume assessment remains crucial, incorporating echocardiography [37, 38], ultrasound [39], and other hemodynamic indices alongside EVLWI. TPTD retains advantages of simplicity and low interobserver variability [40, 41], and with ECBF-adjusted correction, it may be applicable in VV ECMO patients.

However, Bland–Altman analysis revealed wide limits of agreement between TPTD- and CT-derived measurements. Although systematic bias could be reduced after correction for extracorporeal blood flow, these findings indicate substantial variability at the individual patient level. Several mechanisms may account for this discrepancy. Local accumulation of inflammatory cells and edema fluid may alter lung density without proportionally affecting thermodilution-derived measurements. In addition, redistribution of blood from the systemic to the pulmonary circulation may influence indicator dilution kinetics and thereby affect EVLWI estimates. Other conditions that modify thermodilution curves, such as cardiac dysfunction or pleural effusions, may further contribute to the observed variability by altering the mean transit time and downslope time of the indicator curve [16]. Because these factors could not be systematically evaluated in the present dataset, their relative contribution to the differences between CT- and TPTD-derived measurements remains uncertain.

### Limitations

This study has several limitations. First, it was a retrospective single-center analysis, which may limit generalizability. Second, we did not measure lung wet/dry-ratios, which remain the experimental reference standard for quantifying pulmonary edema [16, 42]. Consequently, we cannot determine the absolute accuracy of CT-based or thermodilution-based EVLWI measurements in this cohort. Third, although our ECMO setup was standardized, the correction factor derived here requires external validation. Despite the short interval between CT imaging and TPTD measurement, potential confounders such as fluid administration or vasoactive therapy cannot be completely excluded. In particular, cumulative fluid balance during this interval was not specifically analyzed. Nevertheless, EVLWI typically evolves over several days [43–45], and the marked reduction in bias after correction supports the robustness of our model. Ventilator management may represent another potential confounder in EVLWI measurements obtained by transpulmonary thermodilution. In our cohort, however, ventilatory settings were protocolized and remained unchanged during the interval between transpulmonary thermodilution measurement and CT acquisition, thereby minimizing this potential source of variability. Importantly, prior studies indicate that other TPTD-derived variables such as stroke volume and intrathoracic blood volume index are less affected by ECBF [12, 13]. This observation suggests that the interference observed in our study is largely specific to EVLWI estimation rather than reflecting a generalized limitation of the thermodilution technique during ECMO. In addition, CT scans performed with contrast agents were not excluded. Because contrast media can influence Hounsfield unit measurements, they may theoretically affect the calculated lung weight and, consequently, CT-derived EVLWI estimates. However, in our previous analysis using the same segmentation workflow, contrast administration and segmentation quality did not significantly affect EVLWI estimates [16]. For this reason, we did not perform a separate subgroup analysis in the present study.

## Conclusion

In severe ARDS patients on VV ECMO, TPTD systematically overestimates EVLWI due to ECBF interference. AI-based automated CT segmentation provides a robust reference estimate of pulmonary edema and allows derivation of an ECBF-adjusted correction factor. Application of this correction substantially improves the agreement between TPTD- and CT-derived EVLWI measurements. The resulting correction coefficient of 3.439 ml/kg per liter of extracorporeal blood flow is consistent with previously reported physiological estimates [13]. In clinical practice, EVLWI measurements obtained by transpulmonary thermodilution in VV ECMO patients may therefore be interpreted using an approximate correction factor of 3.0 ml/kg per liter of extracorporeal blood flow.

## Supplementary Information

Supplementary Material 1.

## Data Availability

The datasets analyzed during the current study are available from the corresponding author on reasonable request.

## Abbreviations

AI: Artificial intelligence
ARDS: Acute Respiratory Distress Syndrome
CT: Computed Tomography
ECBF: Extracorporeal Blood Flow
EVLWI: Extravascular Lung Water Index
EVLWICT: Extravascular Lung Water Index in Automatically Segmented CT Scans
EVLWICTman: Extravascular Lung Water Index in Manually Segmented CT Scans
EVLWITPTD: Extravascular Lung Water Index in Transpulmonary Thermodilution
EVLWITPTDcorr: Extravascular Lung Water Index in Transpulmonary Thermodilution after Correction
HU: Hounsfield units
IBW: Ideal Body Weight
LoA: Limit of Agreement
Q: Quartile
SD: Standard Deviation
TPTD: Transpulmonary Thermodilution
VV ECMO: Veno- Venous Extracorporeal Membrane Oxygenation
