# Supplementary material for: Comparative assessment of extravascular lung water index in ARDS patients on veno-venous ECMO: transpulmonary thermodilution versus AI-driven chest CT segmentation
Source: BMC Pulm Med. 2026 Apr 7;26:174. doi: 10.1186/s12890-026-04260-9 (PMC13081323; doi:10.1186/s12890-026-04260-9)
Supplement: Supplementary file 1 — Supplementary Material 1. [file 12890_2026_4260_MOESM1_ESM.docx]

Comparative Assessment of Extravascular Lung Water Index in ARDS Patients on ECMO: Transpulmonary Thermodilution Versus AI-Driven Chest CT Segmentation

- Additional Files -

Matthias Otto, David Mohr, Julia Zimmermann, Nils Rathmann, Christoph Boesing, Manfred Thiel, Joerg Krebs, Thomas Luecke, Patricia R.M. Rocco, Alice Marguerite Conrad

**Institutional management strategy for patients on VV ECMO**

**Transpulmonary Thermodilution**

**Figure S1** Flowchart of the study protocol and patient inclusion

**Table S1** Respiratory mechanics, gas exchange and ventilator settings on the day of the CT scan

**Table S2** Advanced hemodynamic parameters of 64 ARDS patients on VV ECMO on the day of the CT scan

**Table S3**  Laboratory parameters of 64 ARDS patients on VV ECMO on the day of the CT scan

**Table S4** Extravascular lung water index (EVLWI) at different extracorporeal blood flow (ECBF) levels

**Automated versus manual CT quantification of EVLWI**

**Figure S2** Comparison between EVLWI quantified by automated CT scan segmentation and TPTD in Grade 1 ARDS Patients

**Figure S3** Spearman correlation analysis between EVLWI quantified by automated CT scan segmentation and TPTD in Grade 1 ARDS Patients

**Figure S4** Bland-Altman analysis of EVLWI quantified by automated CT scan segmentation and TPTD in Grade 1 ARDS Patients

**Figure S5** Comparison between EVLWI quantified by automated CT scan segmentation and TPTD in Grade 2 ARDS Patients

**Figure S6** Spearman correlation analysis between EVLWI quantified by automated CT scan segmentation and TPTD in Grade 2 ARDS patients

**Figure S7** Bland-Altman analysis between EVLWI quantified by automated CT scan segmentation and TPTD in Grade 2 ARDS patients

**Figure S8** Comparison between EVLWI quantified by automated CT scan segmentation and TPTD in Grade 3 ARDS patients

**Figure S9** Spearman correlation analysis between EVLWI quantified by automated CT scan segmentation and TPTD in Grade 3 ARDS patients

**Figure S10** Bland-Altman analysis of EVLWI quantified by automated CT scan segmentation and TPTD in Grade 3 ARDS patients

**Figure S11** Comparison between EVLWI measured by automated vs. manual CT scan segmentation

**Figure S12** Spearman correlation analysis between EVLWI measured by automated vs.manual CT scan segmentation

**Figure S13** Bland-Altman analysis of EVLWI measured by automated vs. manual CT scan segmentation

**STROBE Statement**

**Institutional management strategy for patients on VV ECMO**

### Indications for VV ECMO

As recommended by current guidelines (1, 2) veno-venous extracorporeal membrane oxygenation (VV ECMO) is initiated in patients with severe acute respiratory distress syndrome (ARDS) as defined by the EOLIA trial eligibility criteria (3). VV ECMO is also considered in patients without clinical improvement despite protective mechanical ventilation after two cycles of prone positioning (3, 4). However, VV ECMO is not initiated in patients under palliative care or those with known end-stage chronic cardiopulmonary failure.

*Veno-venous extracorporeal membrane circuit and management*

VV ECMO is established using a 29-Fr multistage drainage cannula and a 23-Fr venous return cannula (HLS Cannula, Maquet, Rastatt, Germany), inserted via the right femoral and right internal jugular veins, respectively. The extracorporeal circuit includes a magnetically levitated centrifugal pump (Centrimag Circulatory Support System, Abbott GmbH, Wiesbaden, Germany) and a polymethylpentene gas-exchange membrane (PLS System, Maquet, Rastatt, Germany). Blood and sweep gas flows are adjusted to maintain an arterial partial pressure of oxygen (PaO₂) between 65–90 mmHg and an arterial pH of 7.35–7.45 (3).

Mechanical ventilation during ECMO support is initiated in volume-controlled mode with a tidal volume of 2 mL/kg predicted body weight, a respiratory rate of 12 breaths per minute, and a fraction of inspired oxygen (FiO₂) of 40%. Positive end-expiratory pressure (PEEP) is titrated to achieve the lowest elastance of the respiratory system, as previously described (5).

After initial stabilization, a negative fluid balance is targeted using diuretics or continuous renal replacement therapy, and spontaneous breathing is encouraged by gradually tapering analgosedation. A VV ECMO weaning trial is performed when clinically appropriate by reducing the gas flow to 0 L/min for at least 24 hours. ECMO support is discontinued if PaO₂ > 70 mmHg and arterial pH > 7.25 are maintained with FiO₂ < 60% and an inspiratory plateau pressure < 30 cmH₂O (3, 5).

**Transpulmonary Thermodilution**

All patients were equipped with a central venous catheter inserted via the internal jugular vein and a femoral arterial thermodilution catheter (Pulsiocath, Pulsion Medical Systems, Munich, Germany) for advanced hemodynamic monitoring (6). Chest radiography was performed to confirm correct positioning of the central venous catheter, in accordance with institutional protocols. Chest radiography was performed to confirm correct positioning of the central venous catheter, in accordance with institutional protocols.

Transpulmonary thermodilution (TPTD) measurements were performed immediately after patients returned from the CT scan suite to the intensive care unit. Following manufacturer recommendations, three consecutive boluses of 20 mL cold saline (4 °C) were administered via the central venous line to calculate mean values of hemodynamic parameters.

**Figure S1** Flowchart of the study

**
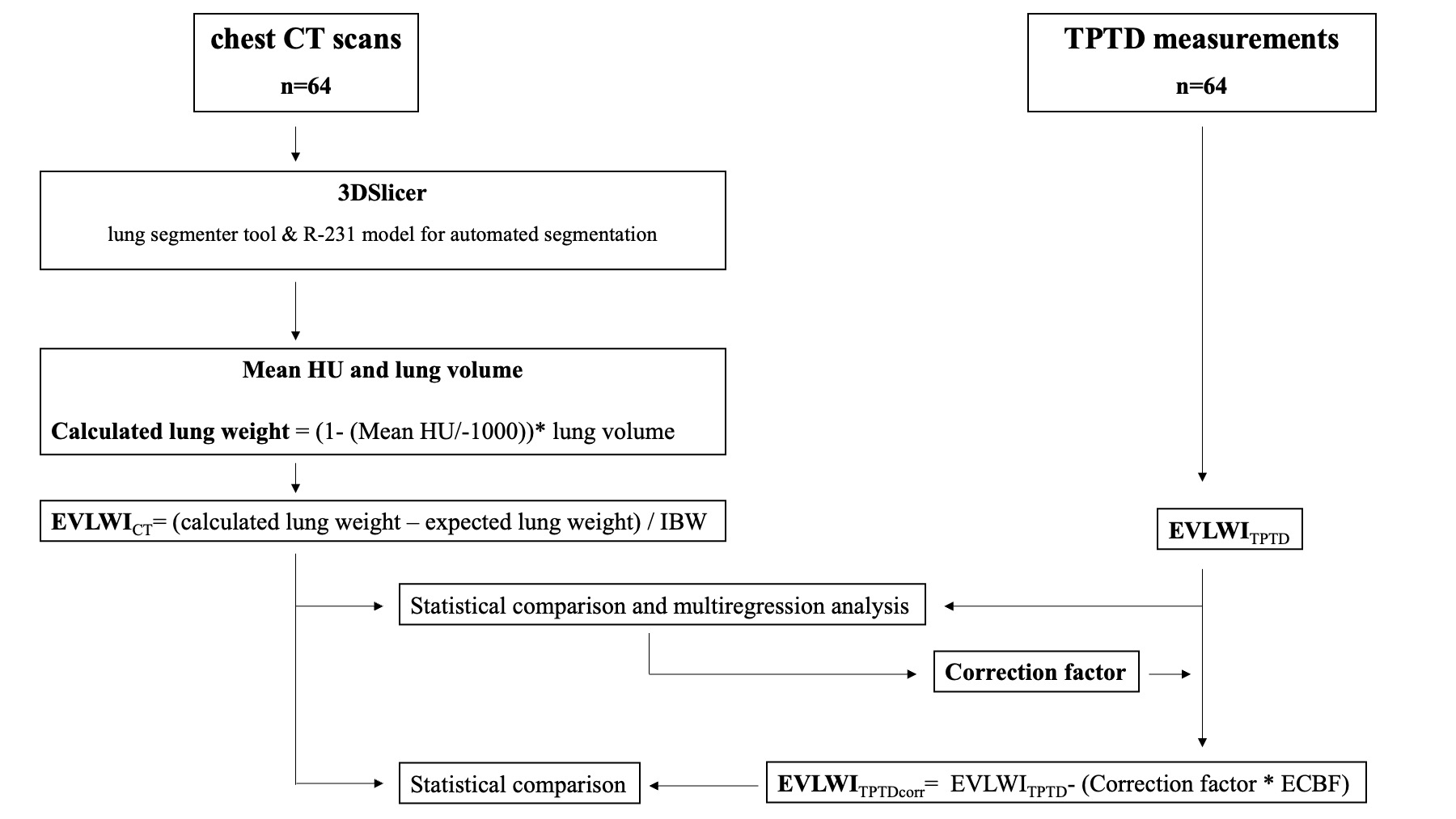
**

CT, *computed tomography*; HU, *Hounsfield units*; EVLWI_CT_, *extravascular lung water index quantified by automated chest CT segmentation*; IBW, *ideal body weight*; TPTD, *transpulmonary thermodilution*; EVLWI_TPTD_, *extravascular lung water index quantified by transpulmonary thermodilution*; ECBF, *extracorporeal blood flow*; EVLWI_TPTDcorr_, *transpulmonary thermodilution-derived extravascular lung water index corrected for extracorporeal blood flow.*

**Table S1** Respiratory mechanics, gas exchange and ventilator settings of 64 ARDS patients on VV ECMO on the day of the CT scan

|  | **Study population**  n = 64 |
| --- | --- |
|  |  |
| Tidal Volume [ml] | 280 ± 110 |
| Respiratory Rate [1/min] | 14 ± 6 |
| Airway Peak Pressure [cmH_2_O] | 28 ± 6 |
| Positive End-Expiratory Pressure [cmH_2_O] | 16 ± 4 |
| Dynamic Compliance of the Respiratory System [ml/cmH_2_O] | 24 ± 15 |
| PaO_2_ [mmHg] | 110 ± 64 |
| PaCO_2_ [mmHg] | 58 ± 13 |
| pHa | 7.3 ± 0.1 |
| ScvO_2_ [%] | 74 ± 9 |
| Lactate [mmol/l] | 2.6 ± 2.9 |
| Ventilator modes:  pressure control – assist control ventilation | 3 |
| biphasic positive airway pressure ventilation | 6 |
| volume-controlled ventilation | 55 |

Data are presented as mean ± SD. Ventilation modes are absolute numbers. PaO_2_, *arterial* *partial pressure of oxygen*; FiO_2_, *fraction of inspired oxygen*; pHa, *arterial pH*; PaCO_2_, *arterial partial pressure of carbon dioxide*; ScvO_2_, *central venous oxygen saturation*

**Table S2** Advanced hemodynamic parameters of 64 ARDS patients on VV ECMO on the day of the CT scan

| **Parameter** | **Study population**  n = 64 |
| --- | --- |
|  |  |
| Heart Rate [1/min] | 102 ± 23 |
| Mean Arterial Pressure [mmHg] | 78 ± 12 |
| Cardiac Stroke Volume [ml] | 75 ± 12 |
| Cardiac Index [l/min/m^2^] | 3.3 ± 1.0 |
| Intrathoracic Blood Volume Index [ml/m²] | 861 ± 314 |
| Systemic Vascular Resistance Index [dyn*s*cm -5*m²] | 1928 ± 794 |
| Vasoactive inotropic score | 13 (4 – 35) |

Data are presented as mean ± SD or median (interquartile range).

**Table S3** Laboratory data of 64 ARDS patients on VV ECMO on the day of the CT scan

| **Parameter** | **Study population**  n = 64 |
| --- | --- |
| Albumin [g/l] | 18 (14-21) |
| Creatinine [mg/dl] | 1.5 (1-2.6) |
| C-Reactive Protein [mg/l] | 217 (106-283) |
| White Blood Cells [10^9^/l] | 11 (9-17) |
| Procalcitonin [µg/l] | 1.9 (0.3-7.5) |
| D-Dimer [mg/l] | 7 (3-14) |

Data are presented as median (interquartile range).

**Table S4** Extravascular lung water index (EVLWI) at different extracorporeal blood flow (ECBF) levels

| **ECBF [l/min]** | **n =** | **EVLWI_CT_**  **[ml/kg]** | **EVLWI_TPTD_**  **[ml/kg]** | **p =** | **EVLWI_TPTDcorr_**  **[ml/kg]** | **p =** |
| --- | --- | --- | --- | --- | --- | --- |
|  |  |  |  |  |  |  |
| 2 – 2.9 | 15 | 5.7 (1.9 – 14.8) | 15.0 (11.0 – 22.3) | 0.002 | 6.0 (4.0 – 13.7) | 0.978 |
| 3 – 3.9 | 14 | 13.4 (6.4 – 16.9) | 19.7 (13.0 – 25.8) | 0.01 | 8.7 (1.1 – 15.1) | 0.391 |
| 4 – 4.9 | 22 | 10.6 (8.0 – 12.5) | 28.6 (21.6 – 31.4) | 0.0002 | 14.0 (7.0 – 17.0) | 0.946 |
| 5 – 6.7 | 13 | 9.2 (7.6 – 12.3) | 26.4 (24.2 – 31.2) | 0.0002 | 8.4 (4.6 – 13.4) | 0.590 |

Comparison of extravascular lung water index (EVLWI) in severe ARDS patients on VV ECMO.

Comparison of EVLWI quantified by three methods: (1) automated chest CT segmentation (EVLWI_CT_), (2) transpulmonary thermodilution without correction (EVLWI_TPTD_), and (3) transpulmonary thermodilution with a correction factor for extracorporeal membrane oxygenation blood flow (EVLWI_TPTDcorr_). The number of measurements (n) corresponds to the respective ECBF level for each patient.
Data are presented as median (interquartile range).

EVLWI_CT_, *extravascular lung water index derived from AI-based automated chest CT segmentation;* EVLWI_TPTD_, *extravascular lung water index derived from transpulmonary thermodilution*; ECBF, *extracorporeal blood flow*; EVLWI_TPTDcorr_, *transpulmonary thermodilution-derived EVLWI corrected for extracorporeal blood flow (ECBF)*

**Automated *versus* Manual CT quantification of extravascular lung water index**

To evaluate the performance of the AI-based algorithm, automated lung segmentation was compared to manual segmentation across all patients. Segmentation quality was assessed using a three-point semi-quantitative scoring system to reflect agreement with manual delineation:

- **Grade 1 (Good):** Automated and manual segmentations are in full agreement.
- **Grade 2 (Moderate):** Differences < 1 cm on more than one slice, or > 1 cm on a single slice.
- **Grade 3 (Poor):** Differences > 1 cm on more than one slice.

Based on this grading system, 30 patients (47%) were classified as **Grade 1 (Good)**, 20 patients (31%) as **Grade 2 (Moderate)**, and 14 patients (22%) as **Grade 3 (Poor)**, indicating that the AI algorithm achieved high or moderate concordance in the majority of cases (7).

**Figure S2** Comparison of EVLWI measured by automated CT segmentation and TPTD in Grade 1 ARDS patients


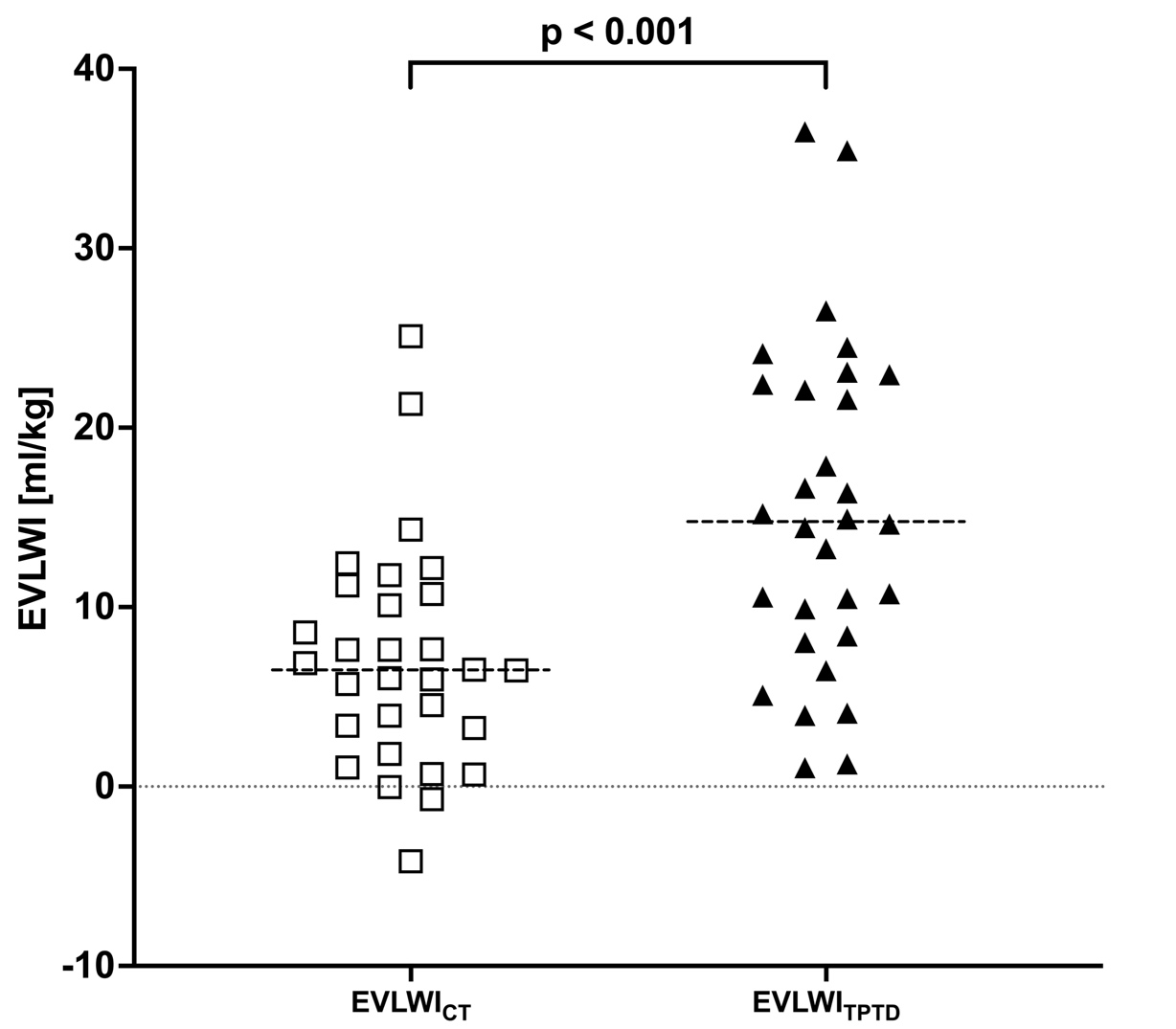


Extravascular lung water index (EVLWI) in severe ARDS patients on VV ECMO, quantified by automated CT scan segmentation (EVLWI_CT_) and TPTD (EVLWI_TPTD_) in Grade 1 patients. The scatter dot plot show the range of values, with dashed horizontal lines representing the medians.

Statistical differences between the measurement modalities are indicated by brackets.

**Figure S3** Spearman correlation analysis between EVLWI quantified by automated CT scan segmentation and TPTD in Grade 1 ARDS patients


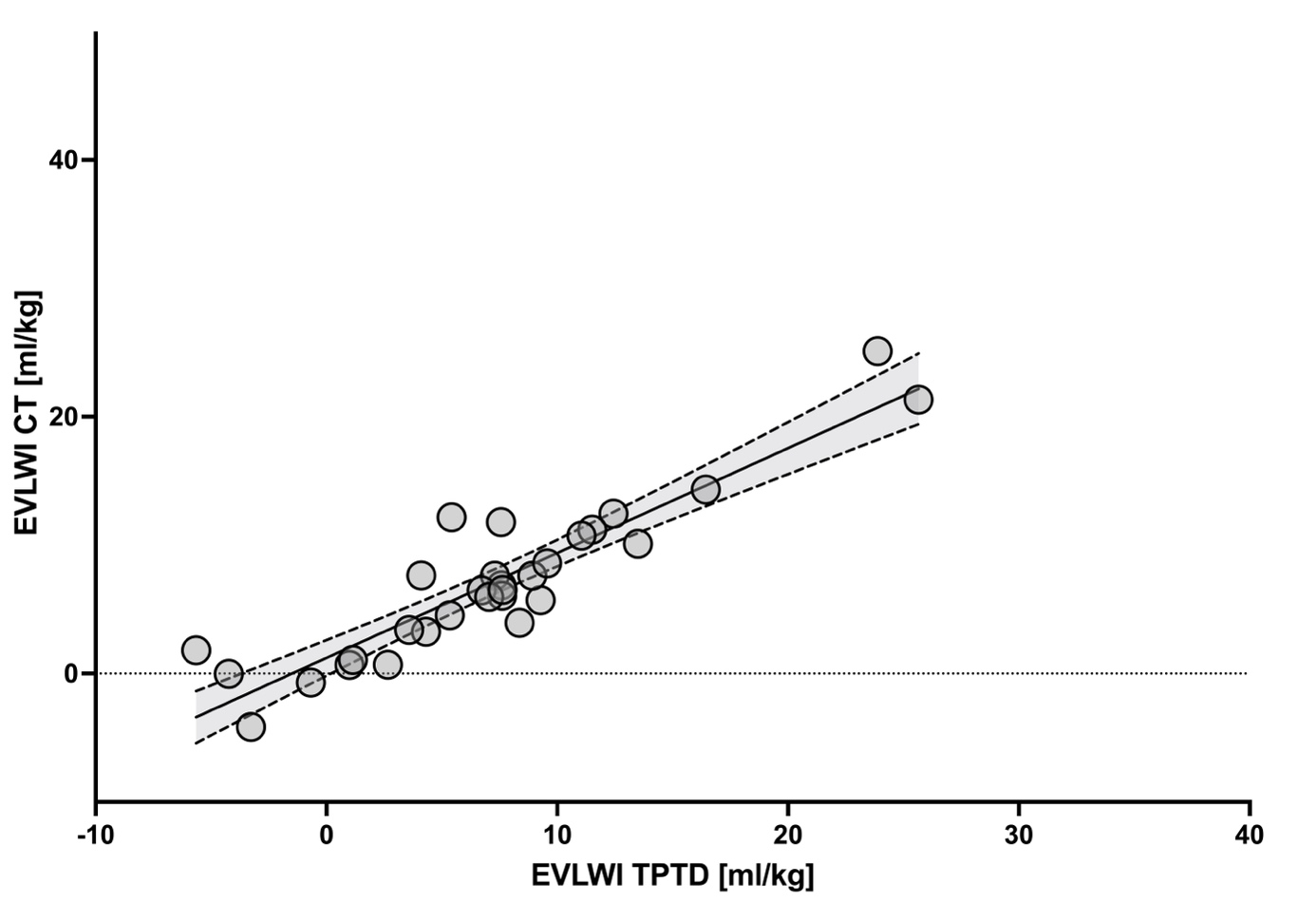


Spearman correlation analysis of extravascular lung water index (EVLWI) quantified by automated CT scan segmentation (EVLWI_CT_) and TPTD (EVLWI_TPTD_) in Grade 1 patients, r= 0.915; p <0.0001; y = 0.817 * x + 1.217

Dashed lines represent the 95% confidence interval.

**Figure S4** Bland-Altman analysis of EVLWI quantified by automated CT scan segmentation and TPTD in Grade 1 ARDS patients


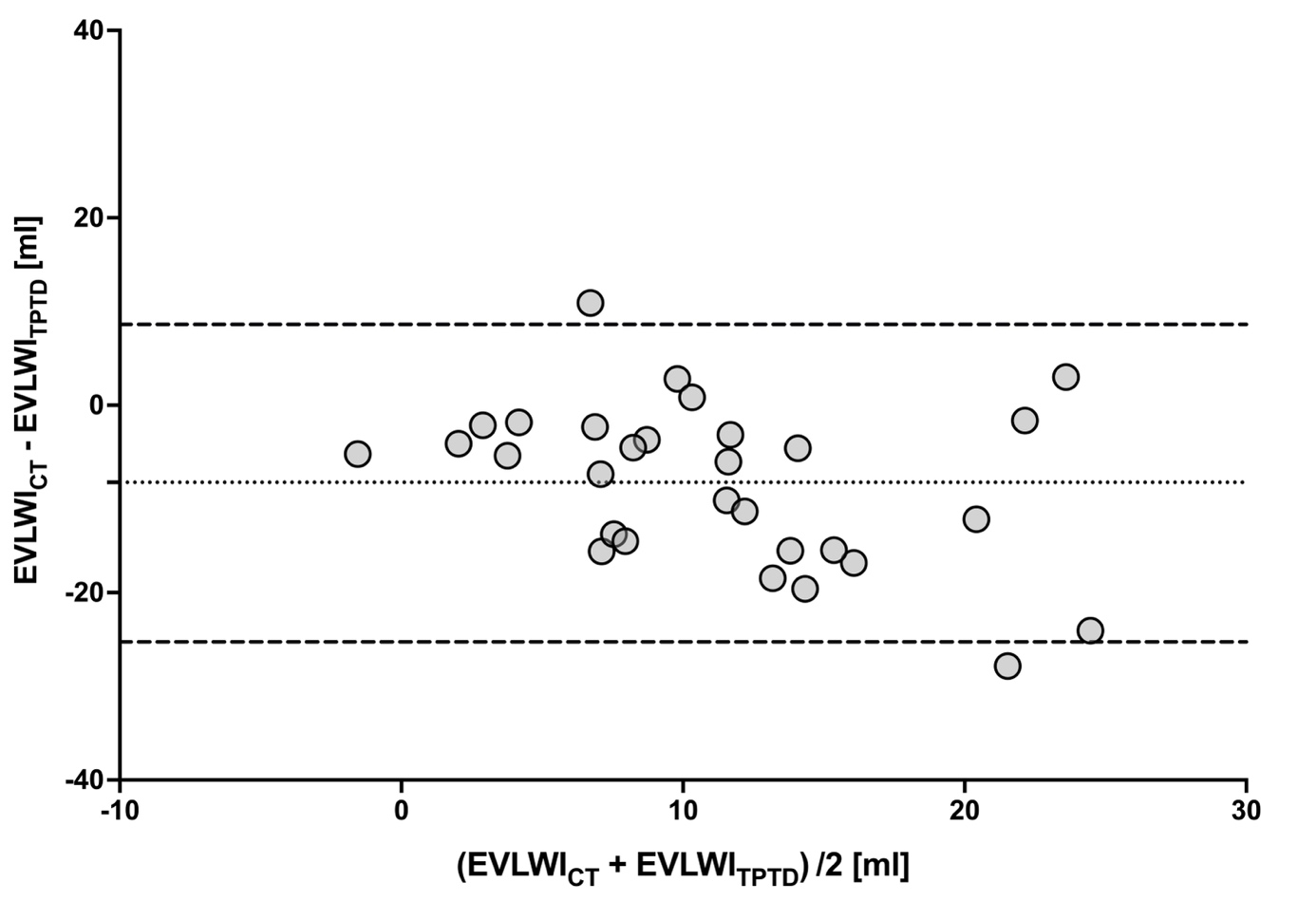


Bland-Altman plot comparing extravascular lung water index between EVLWI quantified by automated CT scan segmentation and TPTD in Grade 1 patients. The dotted line represents the bias (-8.3 ml/kg), while the dashed lines show the limits of agreement (LOA): lower LOA at -25.3 ml/kg and upper LOA at 8.6 ml/kg.

**Figure S5** Comparison between EVLWI quantified by automated CT scan segmentation and TPTD in Grade 2 ARDS patients


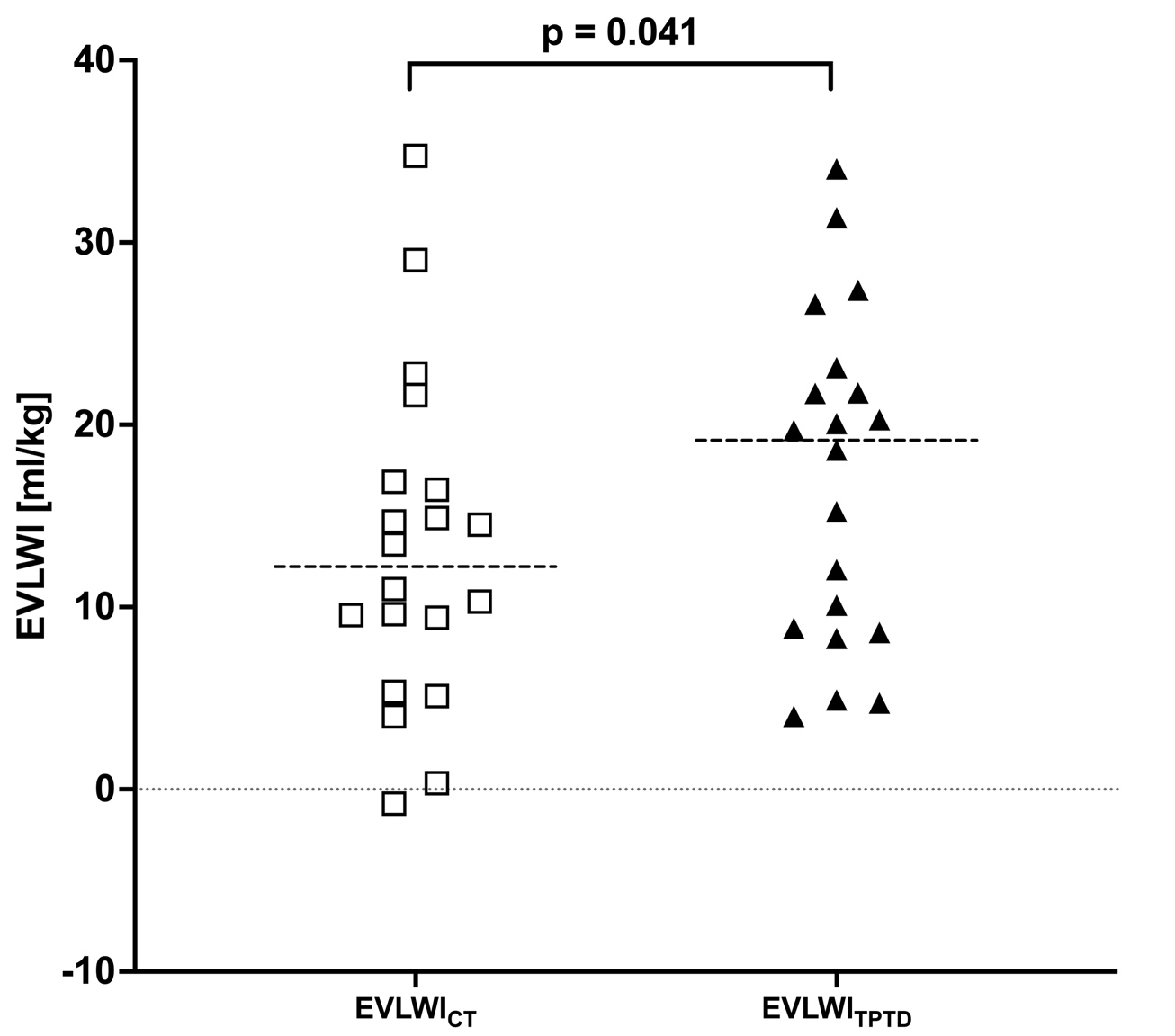


Extravascular lung water index (EVLWI) in severe ARDS patients on VV ECMO, quantified by automated CT scan segmentation (EVLWI_CT_) and TPTD (EVLWI_TPTD_) in Grade 2 patients. The scatter dot plot show the range of values, with dashed horizontal lines representing the medians.

Statistical differences between the measurement modalities are indicated by brackets.

**Figure S6** Spearman correlation analysis between EVLWI quantified by automated CT scan segmentation and TPTD in Grade 2 ARDS patients


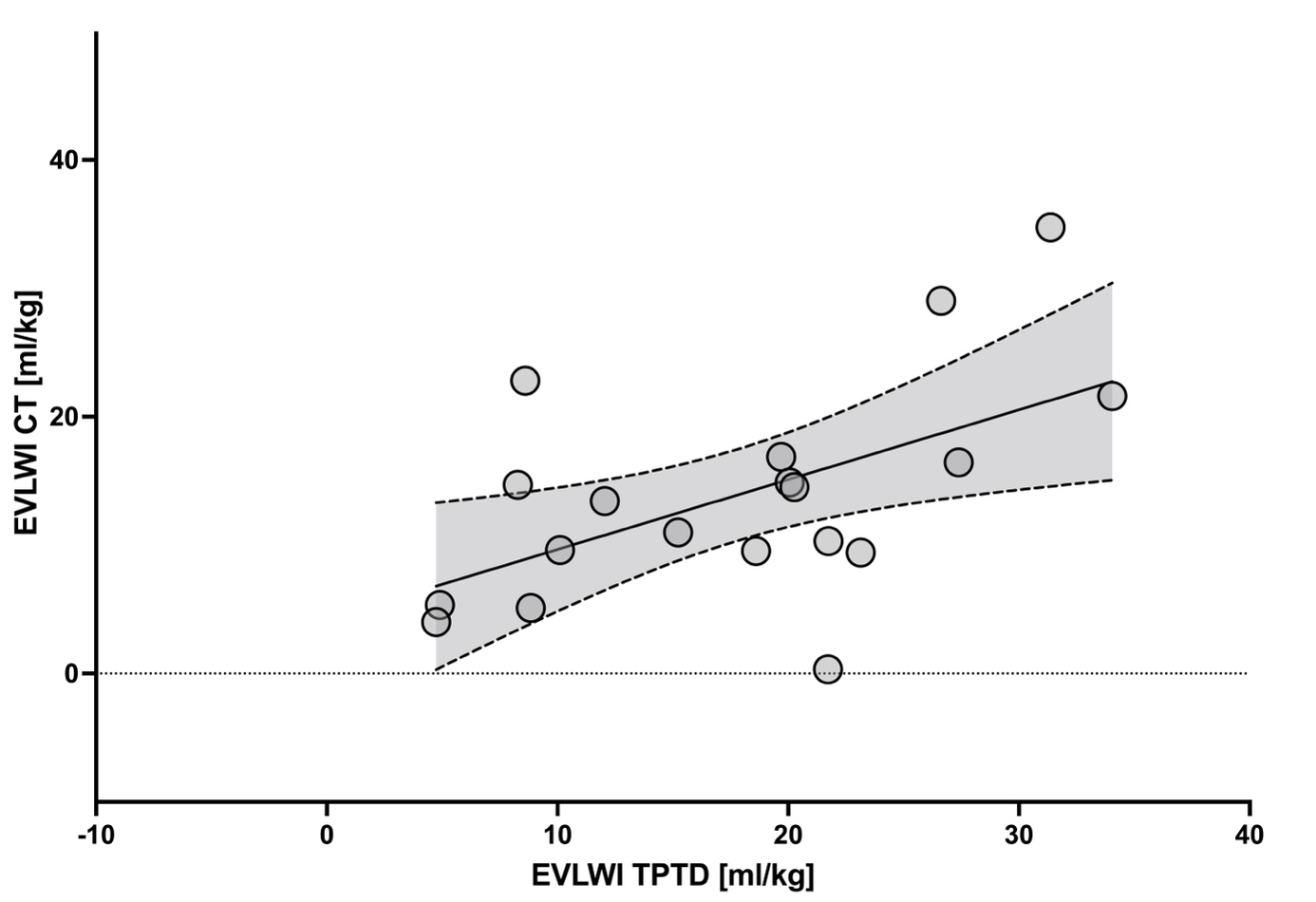


Spearman correlation analysis of extravascular lung water index (EVLWI) quantified by automated CT scan segmentation (EVLWI_CT_) and TPTD (EVLWI_TPTD_) in Grade 2 patients, r= 0.554; p = 0.014; y = 0.652 * x + 10.73

Dashed lines represent the 95% confidence interval.

**Figure S7** Bland-Altman analysis of EVLWI quantified by automated CT scan segmentation and TPTD in Grade 2 ARDS patients

**
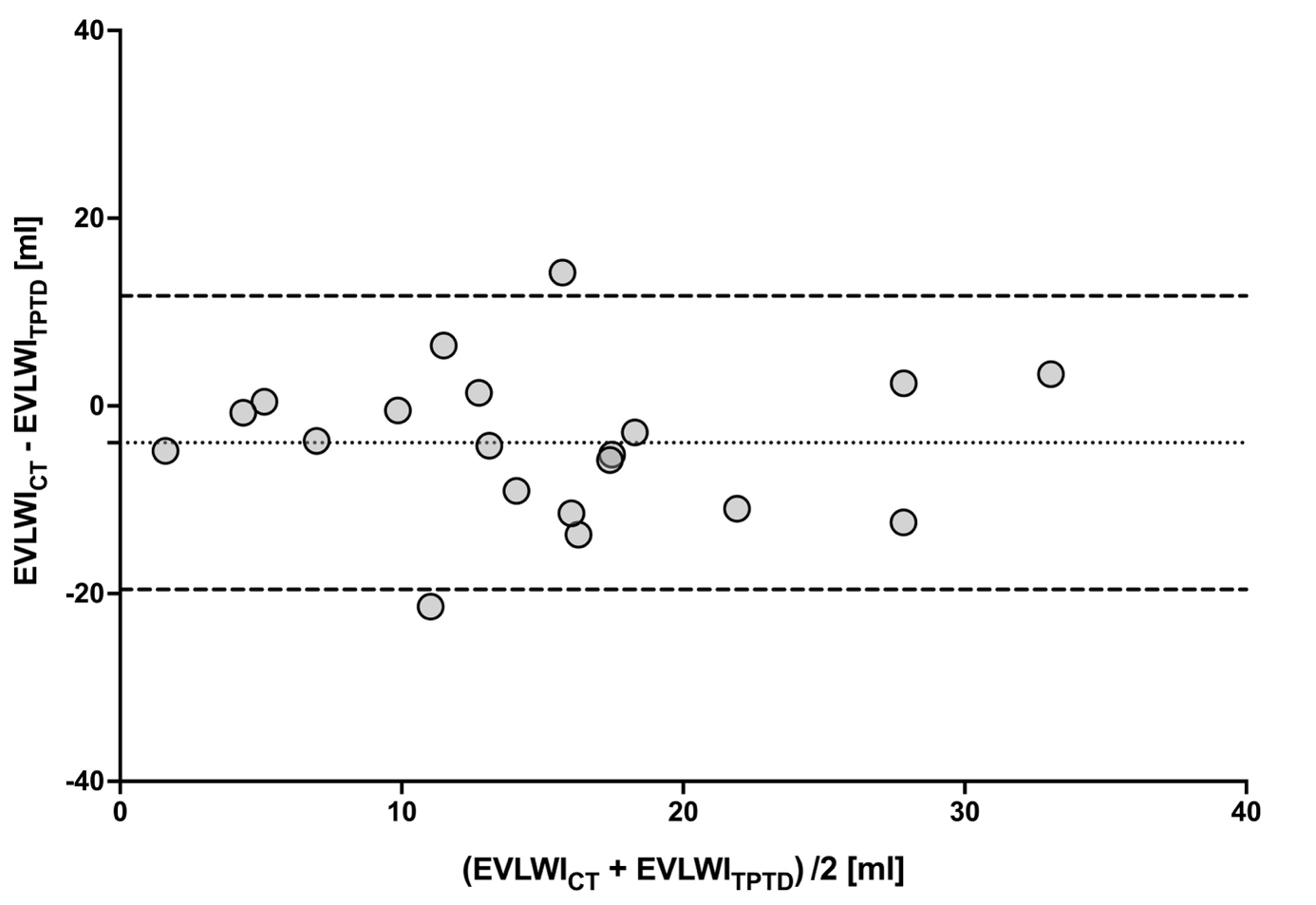
**

Bland-Altman plot comparing extravascular lung water index between EVLWI quantified by automated CT scan segmentation and TPTD in Grade 2 patients. The dotted line represents the bias (-3.9 ml/kg), while the dashed lines show the limits of agreement (LOA): lower LOA at -19.6 ml/kg and upper LOA at 11.7 ml/kg.

**Figure S8** Comparison between EVLWI quantified by automated CT scan segmentation and TPTD in Grade 3 ARDS patients


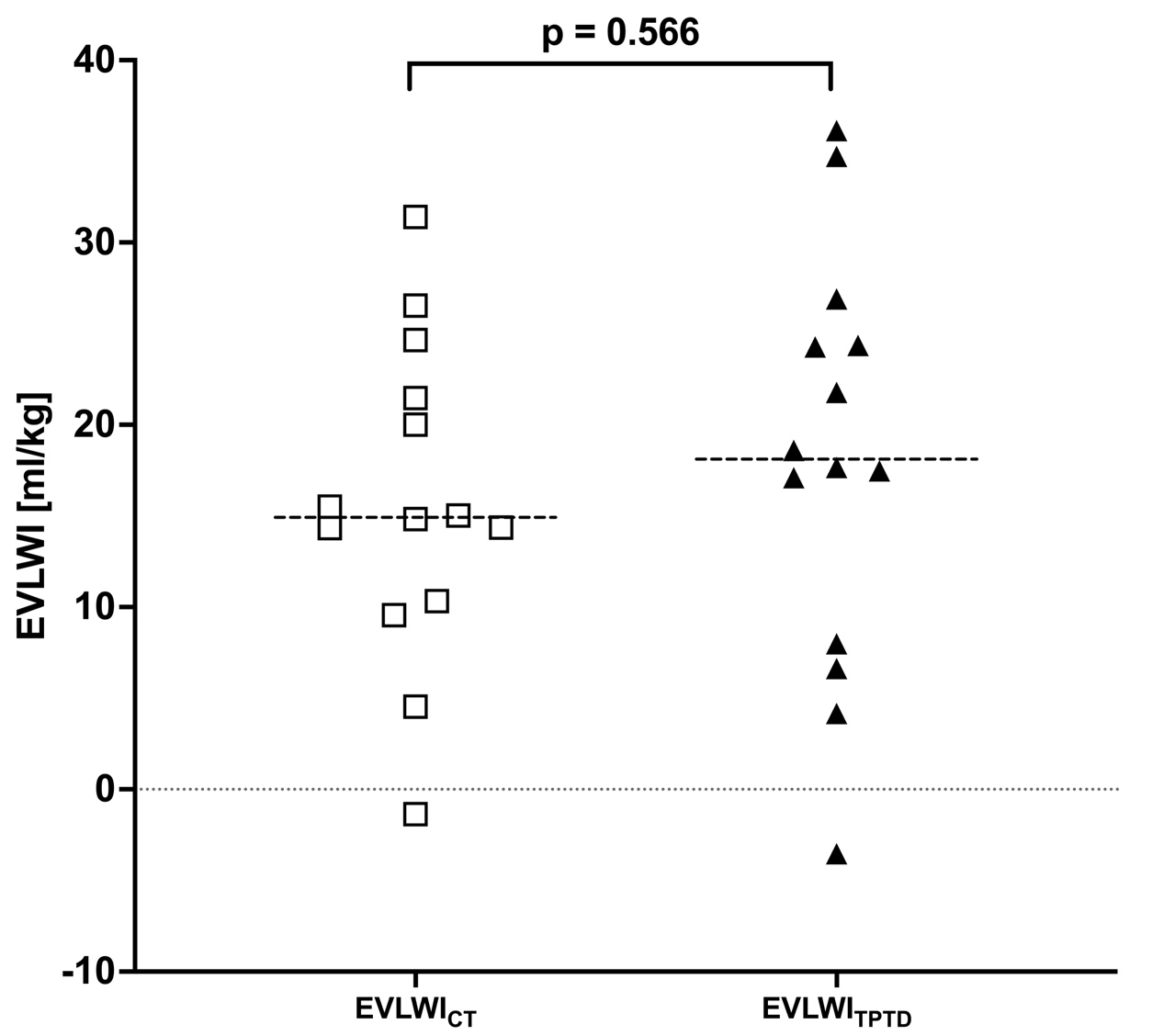


Extravascular lung water index (EVLWI) in severe ARDS patients on VV ECMO, quantified by automated CT scan segmentation (EVLWI_CT_) and TPTD (EVLWI_TPTD_) in Grade 3 patients. The scatter dot plot show the range of values, with dashed horizontal lines representing the medians.

Statistical differences between the measurement modalities are indicated by brackets.

**Figure S9** Spearman correlation analysis between EVLWI quantified by automated CT scan segmentation and TPTD in Grade 3 patients


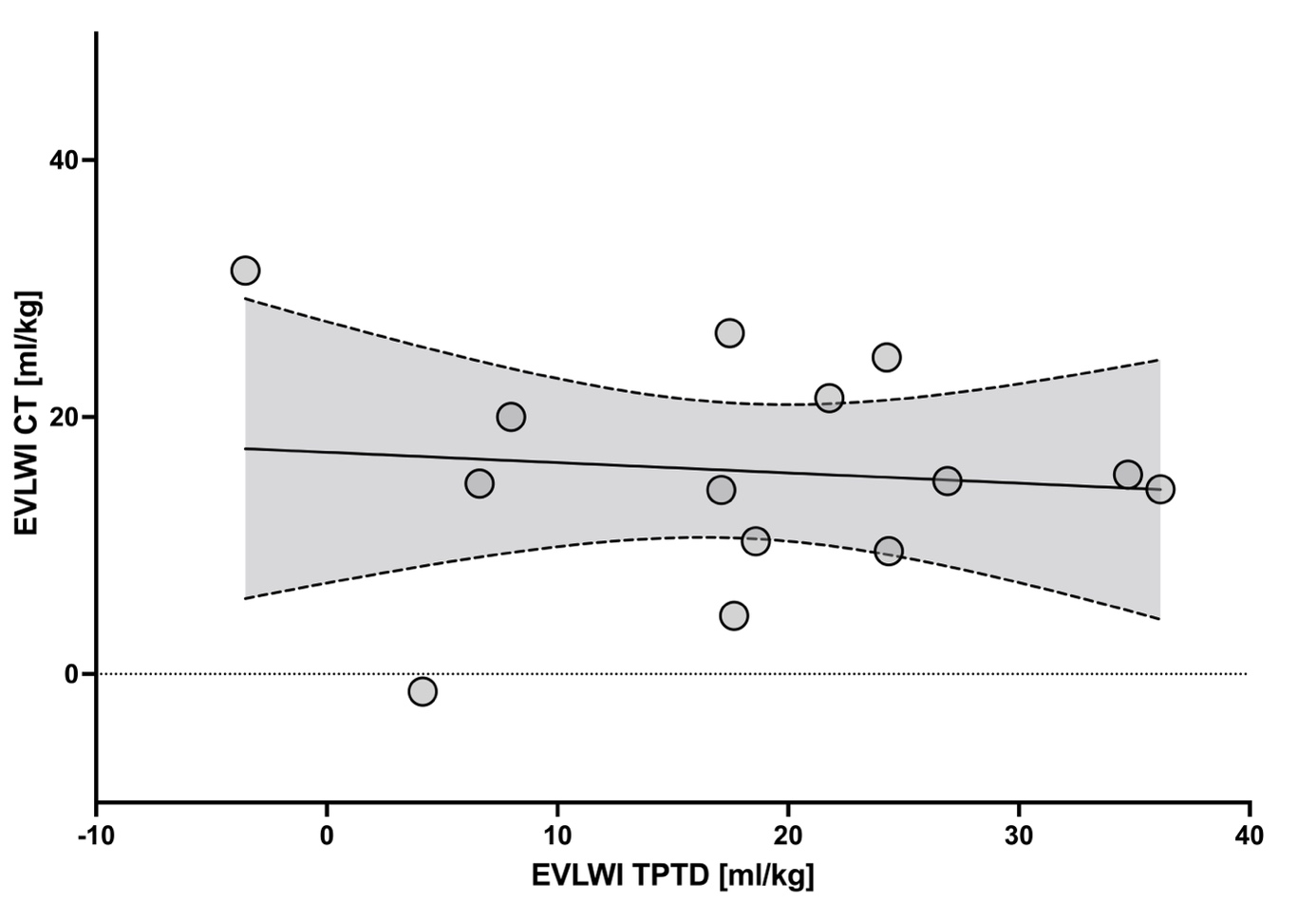


Spearman correlation analysis of extravascular lung water index (EVLWI) quantified by automated CT scan segmentation (EVLWI_CT_) and TPTD (EVLWI_TPTD_) in Grade 3 patients

r= 0.104; p 0 0.722; y = -0.08 * x + 17.26

Dashed lines represent the 95% confidence interval.

**Figure S10** Bland-Altman analysis of EVLWI quantified by automated CT scan segmentation and TPTD in Grade 3 patients

**
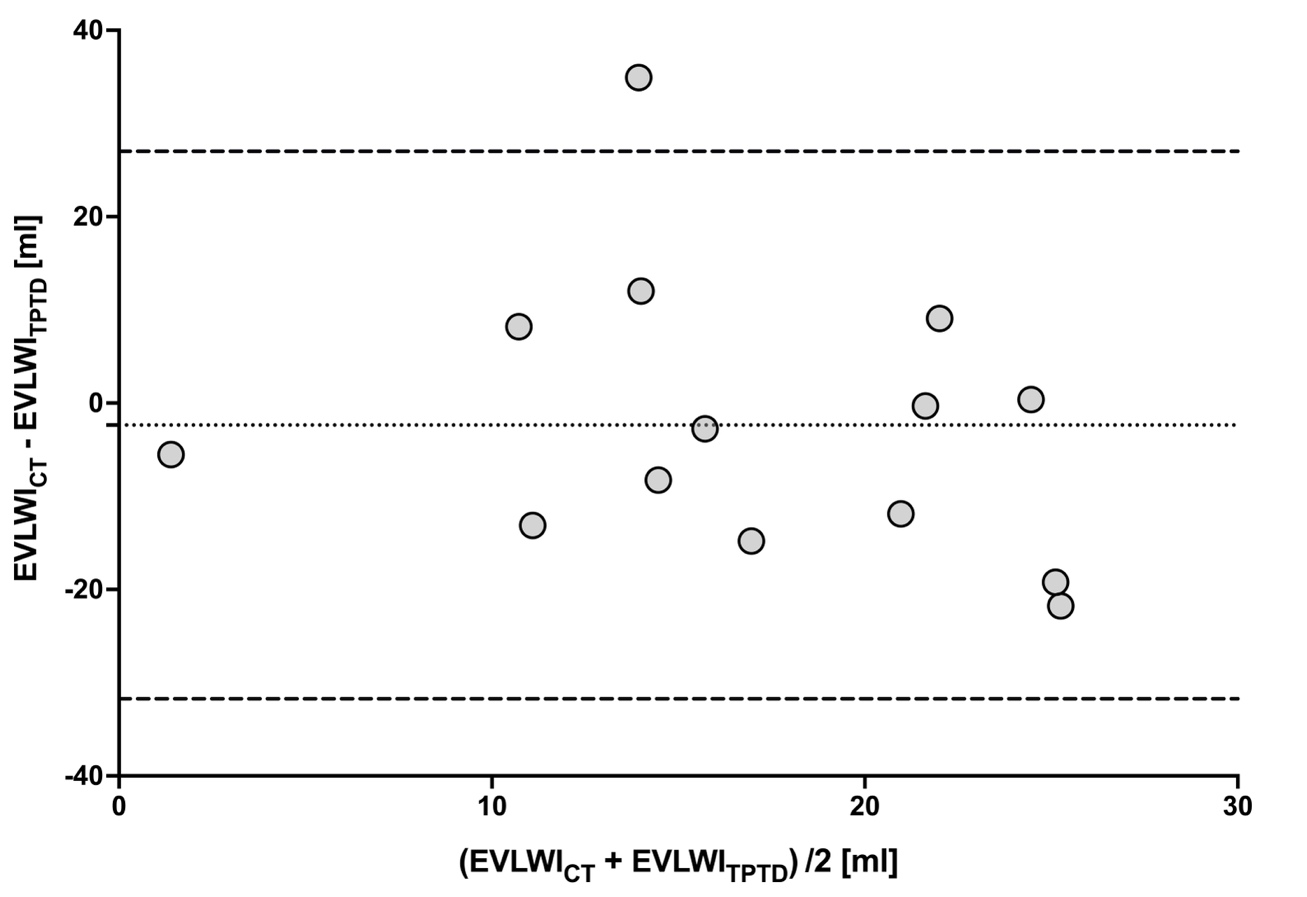
**

Bland-Altman plot comparing extravascular lung water index between EVLWI quantified by automated CT scan segmentation and TPTD in Grade 3 patients. The dotted line represents the bias (-2.4 ml/kg), while the dashed lines show the limits of agreement (LOA): lower LOA at -31.7 ml/kg and upper LOA at 27.0 ml/kg.

EVLWI derived from manual segmentation (EVLWI_CTman_​) was calculated as:

1. $\text{Voxel tissue weight = }\left( \text{1– }\left( \text{Voxel density-1000} \right)/1000 \right)\text{ * Voxel volume}$
2. $\text{C}\text{alculated lung weight = (1- (Mean HU}\text{/}\text{-1000)) * Lung volume}$
3. $\text{E}\text{xpected lung weight (g) = -1806.1 + 1633.7}\text{ }\text{*}\text{ }\text{subject}\text{'}\text{s height (m)}$
4. $\text{EVLWI}_{\text{CTman}}\text{[ml] = (Calculated lung weight - Expected lung weight)/IBW}$

EVLWI_CT_ and EVLWI_CTman_ were examined for Gaussian distribution using Shapiro- Wilk test and then compared using a two-sample *t* test and Mann–Whitney U test, respectively and further analysed using Spearman correlation and Bland-Altman plots. Bias was calculated as the mean difference (8), and 95% limits of agreement (LoA) computed as the mean difference ± 1.96 times the standard deviation.

We found no significant differences (10.2 (5.8/14.8) ml/kg vs 9.6 (5.3/15) ml/kg, p = 0.476) (**Figure S11**) and a high correlation (r= 0.81; p <0.001) (**Figure S12**) between EVLWI_CT_ and EVLWI_CTman_. Bland Altmann analysis showed a bias of 0.5 ml/kg, with a lower level of agreement of – 10.1 ml/kg and an upper level of agreement of 11.1 ml/kg (**Figure S13)**.

**Figure S11** Comparison between EVLWI measured by automated and manual CT scan segmentation


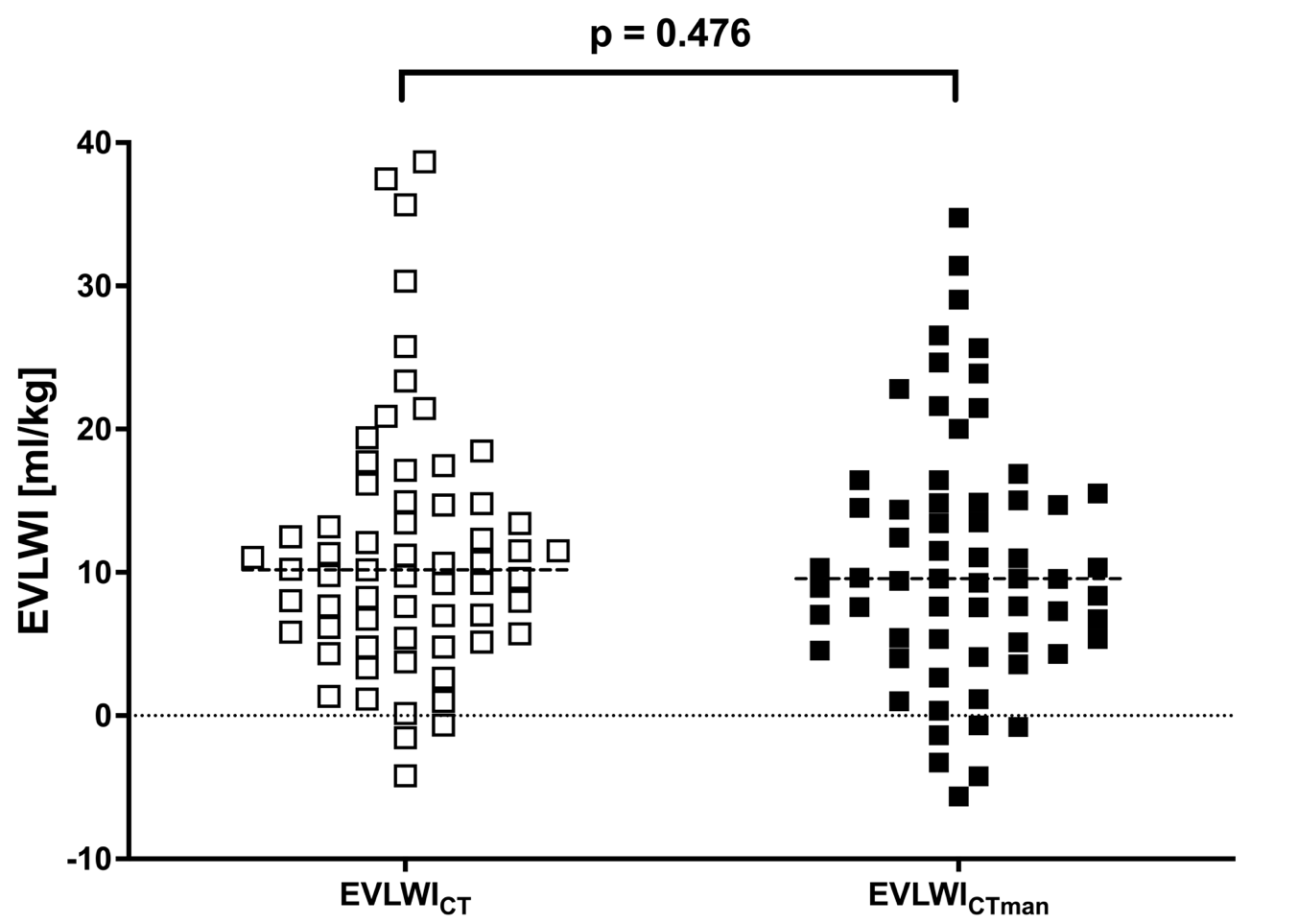


Extravascular lung water index (EVLWI) in severe ARDS patients on VV ECMO, quantified by automated (EVLWI_CT_) and manual (EVLWI_CTman_) chest CT scan segmentation. The scatter dot plot show the range of values, with dashed horizontal lines representing the medians.

Statistical differences between the measurement modalities are indicated by brackets.

**Figure S12** Spearman correlation analysis between EVLWI measured by automated and manual CT scan segmentation

**
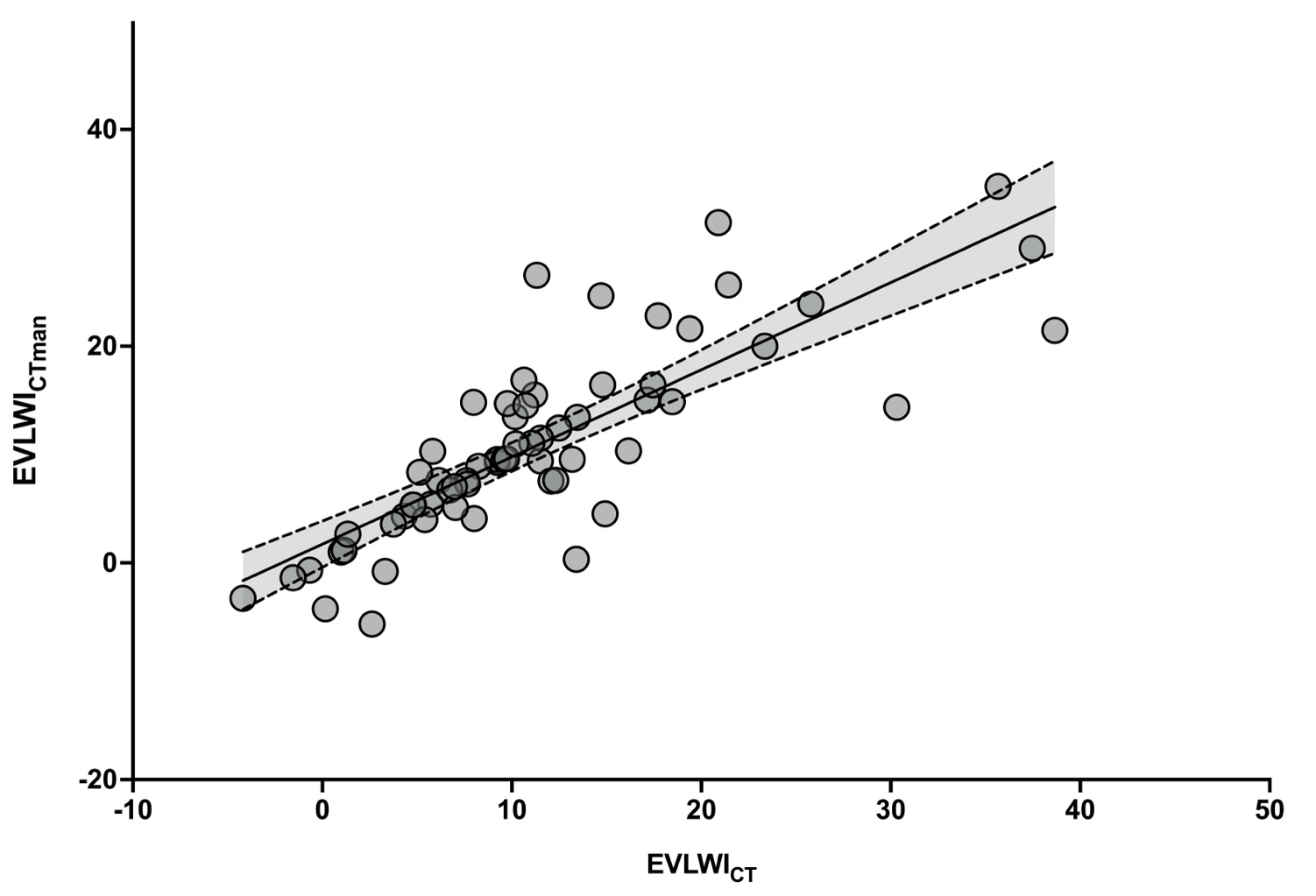
**

Spearman correlation analysis of extravascular lung water index (EVLWI) quantified by automated (EVLWI_CT_) and manual (EVLWI_CTman_) chest CT scan segmentation r= 0.81;

p <0.001; y = 0.8047 * x + 1.73

Dashed lines represent the 95% confidence interval.

**Figure S13** Bland-Altman analysis of EVLWI measured by automated and manual CT scan segmentation

**
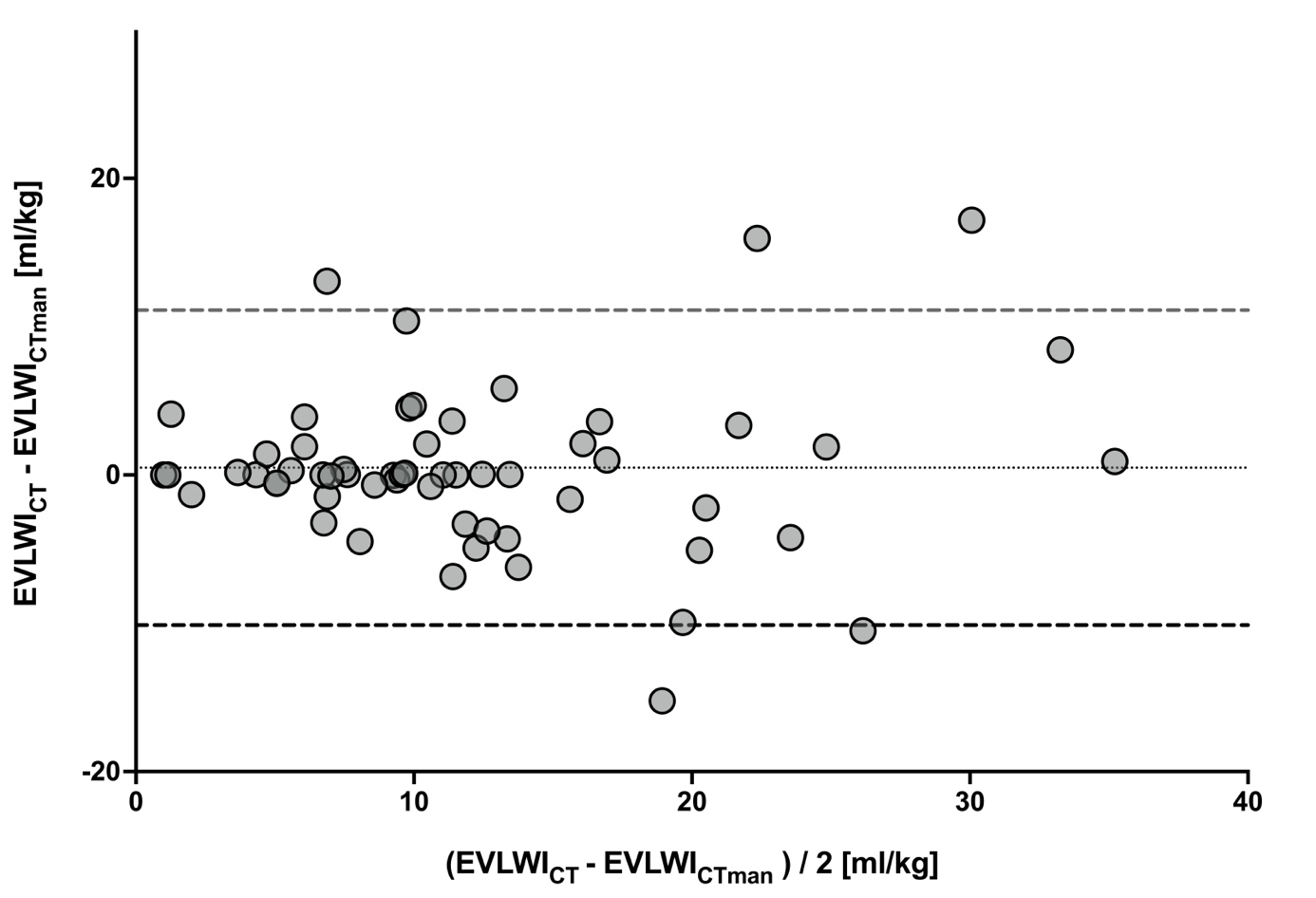
**

Bland-Altman plot comparing extravascular lung water index quantified by automated (EVLWI_CT_) and manual (EVLWI_CTman_) chest CT scan segmentation. The dotted line represents the bias (0.5 ml/kg), while the dashed lines show the limits of agreement (LOA): lower LOA at -10.1 ml/kg and upper LOA at 11.1 ml/kg.

**STROBE Statement**

|  | Item No | Recommendation | Page No |
| --- | --- | --- | --- |
| **Title and abstract** | 1 | (*a*) Indicate the study’s design with a commonly used term in the title or the abstract |  |
|  |  | (*b*) Provide in the abstract an informative and balanced summary of what was done and what was found | 2 |
| Introduction | | | |
| Background/rationale | 2 | Explain the scientific background and rationale for the investigation being reported | 4 |
| Objectives | 3 | State specific objectives, including any prespecified hypotheses | 5 |
| Methods | | | |
| Study design | 4 | Present key elements of study design early in the paper | 5 |
| Setting | 5 | Describe the setting, locations, and relevant dates, including periods of recruitment, exposure, follow-up, and data collection | 5 |
| Participants | 6 | (*a*) Give the eligibility criteria, and the sources and methods of selection of participants. Describe methods of follow-up | 5-6 |
|  |  | (*b*) For matched studies, give matching criteria and number of exposed and unexposed |  |
| Variables | 7 | Clearly define all outcomes, exposures, predictors, potential confounders, and effect modifiers. Give diagnostic criteria, if applicable | 5-8 |
| Data sources/ measurement | 8* | For each variable of interest, give sources of data and details of methods of assessment (measurement). Describe comparability of assessment methods if there is more than one group | 6-8 |
| Bias | 9 | Describe any efforts to address potential sources of bias | 5-6 |
| Study size | 10 | Explain how the study size was arrived at | 8 |
| Quantitative variables | 11 | Explain how quantitative variables were handled in the analyses. If applicable, describe which groupings were chosen and why | 8-9 |
| Statistical methods | 12 | (*a*) Describe all statistical methods, including those used to control for confounding | 8-9 |
|  |  | (*b*) Describe any methods used to examine subgroups and interactions |  |
|  |  | (*c*) Explain how missing data were addressed |  |
|  |  | (*d*) If applicable, explain how loss to follow-up was addressed |  |
|  |  | (*e*) Describe any sensitivity analyses |  |
| Results | | |  |
| Participants | 13* | (a) Report numbers of individuals at each stage of study—eg numbers potentially eligible, examined for eligibility, confirmed eligible, included in the study, completing follow-up, and analysed | 9 |
|  |  | (b) Give reasons for non-participation at each stage |  |
|  |  | (c) Consider use of a flow diagram |  |
| Descriptive data | 14* | (a) Give characteristics of study participants (eg demographic, clinical, social) and information on exposures and potential confounders | Table 1, Additional Files |
|  |  | (b) Indicate number of participants with missing data for each variable of interest | Not applicable |
|  |  | (c) Summarise follow-up time (eg, average and total amount) | Not applicable |
| Outcome data | 15* | Report numbers of outcome events or summary measures over time | 9-10 |

| Main results | 16 | (*a*) Give unadjusted estimates and, if applicable, confounder-adjusted estimates and their precision (eg, 95% confidence interval). Make clear which confounders were adjusted for and why they were included | 9-10 |
| --- | --- | --- | --- |
|  |  | (*b*) Report category boundaries when continuous variables were categorized |  |
|  |  | (*c*) If relevant, consider translating estimates of relative risk into absolute risk for a meaningful time period |  |
| Other analyses | 17 | Report other analyses done—eg analyses of subgroups and interactions, and sensitivity analyses | Additional Files |
| Discussion | | | |
| Key results | 18 | Summarise key results with reference to study objectives | 10 |
| Limitations | 19 | Discuss limitations of the study, taking into account sources of potential bias or imprecision. Discuss both direction and magnitude of any potential bias | 12-13 |
| Interpretation | 20 | Give a cautious overall interpretation of results considering objectives, limitations, multiplicity of analyses, results from similar studies, and other relevant evidence | 13 |
| Generalisability | 21 | Discuss the generalisability (external validity) of the study results | 13 |
| Other information | | | |
| Funding | 22 | Give the source of funding and the role of the funders for the present study and, if applicable, for the original study on which the present article is based | 15 |

**Abbreviations:**

ARDS acute respiratory distress syndrome

CT computed tomography

ECBF extracorporeal blood flow

EVLWI extravascular lung water index

EVLWI_CT_ extravascular lung water index in automatically segmented CT scans

EVLWI_CTman_ extravascular lung water index in manually segmented CT scans

EVLWI_TPTD_ extravascular lung water index in transpulmonary thermodilution

EVLWI_TPTDcorr_ extravascular lung water index in transpulmonary thermodilution after correction

FiO_2_ fraction of inspired oxygen

HU Hounsfield unit

IBW ideal body weight

LoA Limit of agreement

pHa arterial potential of hydrogen

PaCO_2_ arterial partial pressure of carbon dioxide

PaO_2_ arterial partial pressure of oxygen

TPTD transpulmonary thermodilution

ScvO_2_ central venous oxygen saturation

VV ECMO veno- venous extracorporeal membrane oxygenation

**References:**

1. Grasselli G, Calfee CS, Camporota L, Poole D, Amato MBP, Antonelli M, et al. ESICM guidelines on acute respiratory distress syndrome: definition, phenotyping and respiratory support strategies. Intensive Care Med. 2023;49(7):727-59.

2. Tonna JE, Abrams D, Brodie D, Greenwood JC, Rubio Mateo-Sidron JA, Usman A, et al. Management of Adult Patients Supported with Venovenous Extracorporeal Membrane Oxygenation (VV ECMO): Guideline from the Extracorporeal Life Support Organization (ELSO). ASAIO J. 2021;67(6):601-10.

3. Combes A, Hajage D, Capellier G, Demoule A, Lavoue S, Guervilly C, et al. Extracorporeal Membrane Oxygenation for Severe Acute Respiratory Distress Syndrome. N Engl J Med. 2018;378(21):1965-75.

4. Schmidt M, Pham T, Arcadipane A, Agerstrand C, Ohshimo S, Pellegrino V, et al. Mechanical Ventilation Management during Extracorporeal Membrane Oxygenation for Acute Respiratory Distress Syndrome. An International Multicenter Prospective Cohort. Am J Respir Crit Care Med. 2019;200(8):1002-12.

5. Graf PT, Boesing C, Brumm I, Biehler J, Müller KW, Thiel M, et al. Ultraprotective versus apneic ventilation in acute respiratory distress syndrome patients with extracorporeal membrane oxygenation: a physiological study. J Intensive Care. 2022;10(1):12.

6. Cecconi M, De Backer D, Antonelli M, Beale R, Bakker J, Hofer C, et al. Consensus on circulatory shock and hemodynamic monitoring. Task force of the European Society of Intensive Care Medicine. Intensive Care Med. 2014;40(12):1795-815.

7. Conrad AM, Zimmermann J, Mohr D, Froelich MF, Hertel A, Rathmann N, et al. Quantification of pulmonary edema using automated lung segmentation on computed tomography in mechanically ventilated patients with acute respiratory distress syndrome. Intensive Care Med Exp. 2024;12(1):95.

8. Bland JM, Altman DG. Statistical methods for assessing agreement between two methods of clinical measurement. Lancet. 1986;1(8476):307-10.
